# Lipoprotein docosapentaenoic acid is associated with serum matrix metalloproteinase-9 concentration

**DOI:** 10.1186/1476-511X-4-8

**Published:** 2005-04-13

**Authors:** Tiina Solakivi, Olli Jaakkola, Anne Kalela, Mari Pispa, Anne Salomäki, Terho Lehtimäki, Matti Höyhtyä, Hannu Jokela, Seppo T Nikkari

**Affiliations:** 1 Department of Medical Biochemistry, Medical School, University of Tampere, Tampere, Finland; 2 Institute of Medical Technology, University of Tampere, Tampere, Finland; 3 Department of Clinical Chemistry, Tampere University Hospital, Tampere, Finland; 4 Laboratory of Atherosclerosis Genetics, Department of Clinical Chemistry, Medical School, University of Tampere, Tampere, Finland; 5 Medix Biochemica, Kauniainen, Finland

## Abstract

**Background:**

Polyunsaturated fatty acids (PUFA) are thought to play important roles in inflammation. The n-3 series is considered as anti-inflammatory, and some studies have reported increased plasma n-3 polyunsaturated fatty acid pattern in chronic inflammatory conditions. In this study we sought to clarify relationships of the levels of arachidonic acid and the polyunsaturated n-3 fatty acid compositions of isolated LDL, HDL_2 _and HDL_3 _particles with matrix metalloproteinase-9 (MMP-9), a marker of inflammation.

**Results:**

The subjects were divided into two groups: those with lower and those with higher than the median serum MMP-9 concentration. In all lipoprotein fractions, the mean percentage of docosapentaenoic acid (C22:5n-3) was higher in the group of subjects with higher MMP-9 level than in those with lower serum MMP-9 concentration (P < 0.01 for all). Likewise, the ratio of docosapentaenoic acid to arachidonic acid (C20:4n-6) was higher in the subjects with higher MMP-9 compared with the lower MMP-9 group (P < 0.001 for all).

**Conclusion:**

So far, the evidence for an anti-inflammatory role of the n-3 PUFA has come from dietary interventions. Our results were obtained from a free-living population and indicate that there is a positive correlation between n-3 docosapentaenoic acid and MMP-9. What had triggered the rise in MMP-9 is not known, since serum level of MMP-9 is raised in many inflammatory conditions. These findings may indicate an increased biosynthesis of n-3 polyunsaturated fatty acids in subclinical inflammation.

## Background

As a consequence of arterial inflammation, serum MMP-9 is increased in primary arteritis [1,2] and in severe CAD [3]. In addition to these processes, elevated serum MMP-9 has been reported in cancer [4], asthma [5] and rheumatoid arthritis [6]. Thus, in inflammation in general there is a rise of serum MMP-9 concentration.

Polyunsaturated fatty acids (PUFA) may play an important role in inflammation. The two classes of polyunsaturated fatty acids (PUFA), the n-6 and n-3 series have opposing physiological functions. Arachidonic acid (20:4n-6), a metabolite of linoleic acid (18:2n-6), is the substrate of cyclooxygenases and lipoxygenases in the production of potent inflammatory eicosanoids. The polyunsaturated fatty acids of the n-3 series (eicosapentaenoic acid 20:5n-3, docosapentaenoic acid 22:5n-3, and docosahexaenoic acid 22:6n-3) in turn inhibit the synthesis of these mediators and produce eicosanoids with much weaker effects [7,8]. As outlined above, the n-3 series is considered as anti-inflammatory.

Low rates of CHD were found in Greenland Eskimos who are exposed to a diet rich in n-3 fatty acid fish oil [9]. Such diets have also been suggested to reduce the risk of inflammatory bowel disease [10]. Interestingly, some studies have reported increased plasma n-3 polyunsaturated fatty acid pattern in chronic inflammatory bowel diseases, such as Crohn's disease and ulcerative colitis [11,12]. In fact, significantly higher levels of docosapentaenoic acid (22:5n-3) and lower levels of arachidonic acid (20:4n-6) have been reported in serum of patients with long-standing Crohn's disease in comparison with controls [13]. Moreover, ileal and colonic fatty acids profiles in these patients show a substantial increase of the highly polyunsaturated fatty acids [14]. Patients with Crohn's disease have also increased percentage of n-3 PUFA in peripheral blood monocytes [15]. Rats with experimental ulcerative colitis have increased n-3 fatty acids in colon mucosa [16]. These findings were suggested to indicate an increased biosynthesis of polyunsaturated fatty acids in inflammation due to increased activity in the desaturase/elongation enzymes by hitherto unknown mechanisms. With more severe inflammation the essential fatty acids that are precursors to the polyunsaturated fatty acids are reduced, as is observed in patients with rheumatoid arthritis [17] and active colitis ulcerosa [11].

In this study we sought to clarify relationships of the levels of arachidonic acid and the polyunsaturated n-3 fatty acid compositions of isolated LDL, HDL_2 _and HDL_3 _particles with MMP-9, a marker of inflammation.

## Results

The subjects were divided into two groups: those with higher and those with lower MMP-9 levels than the median of 43 μg/l. The characteristics of the groups and the arachidonic acid, eicosapentaenoic acid, docosapentaenoic acid and docosahexaenoic acid compositions of LDL, HDL_2 _and HDL_3 _are shown in Tables I and II, respectively. The background characteristics of the two groups were very similar. The gender distribution was the same in both groups. Mean values for age, BMI, total cholesterol, triacylglycerol HDL or LDL cholesterol did not differ between the two groups. Thus, these background variables were not associated with serum MMP-9 concentrations.

**Table 1:** Background characteristics of the MMP-9 subgroups^*a*^

	Group I^*b *^n = 29	Group II^*c *^n = 29	Significance^*d *^(p-value)
Sex (female/male)	16/13	16/13	1.000000
MMP9 (μg/l)	32.8 ± 5.1	53.8 ± 8.4	0.000000
Age (years)	38.6 ± 10.4	39.7 ± 11.1	0.70
BMI (kg/m^2^)	24.9 ± 4.0	23.9 ± 3.1	0.32
Cholesterol (mmol/l)	5.44 ± 0.81	5.48 ± 1.13	0.90
Triacylglycerol (mmol/l)	1.26 ± 0.66	1.44 ± 1.26	0.51
HDL-cholesterol (mmol/l)	1.65 ± 0.34	1.64 ± 0.42	0.90
LDL-cholesterol (mmol/l)	3.22 ± 0.77	3.25 ± 1.11	0.90
ApoA-I (g/l)	1.61 ± 0.21	1.56 ± 0.21	0.47
ApoB (g/l)	0.90 ± 0.20	0.89 ± 0.26	0.76
fB-Glucose (mmol/l)	4.5 ± 0.6	4.4 ± 0.5	0.36

^*a*^Means ± SD are given; ^*b *^MMP-9 <43 ug/l; ^*c *^MMP-9 >43 ug/l; ^*d*^Mann-Whitney U-test

**Table 2:** Percentages of PUFA in total fatty acids from separated lipoproteins in the MMP-9 subgroups^*a*^

PUFAb	Group I^*c *^n = 29	Group II^*d *^n = 29	Significance^*e *^(p-value)
**Arachidonic Acid**			
LDL-particles	5.61 ± 1.11	5.25 ± 0.87	0.25
HDL2-particles	7.02 ± 1.24	6.70 ± 1.18	0.43
HDL3-particles	7.86 ± 1.46	7.42 ± 1.13	0.28
			
**Eicosapentaenoic Acid**			
LDL-particles	1.13 ± 0.39	1.25 ± 0.74	0.99
HDL2-particles	1.24 ± 0.40	1.37 ± 0.78	0.98
HDL3-particles	1.38 ± 0.43	1.50 ± 0.87	0.86
			
**Docosapentaenoic Acid**			
LDL-particles	0.39 ± 0.08	0.47 ± 0.09	0.0015
HDL2-particles	0.63 ± 0.14	0.74 ± 0.14	0.0057
HDL3-particles	0.69 ± 0.15	0.80 ± 0.15	0.0080
			
**Docosahexaenoic Acid**			
LDL-particles	2.13 ± 0.41	2.05 ± 0.62	0.35
HDL_2_-particles	3.49 ± 0.63	3.27 ± 0.92	0.16
HDL_3_-particles	3.66 ± 0.70	3.47 ± 0.97	0.21

^*a*^Means ± SD are given; ^*b*^PUFA, polyunsaturated fatty acids; ^*c*^MMP-9 <43 ug/l;

^*d*^MMP-9 >43 ug/l; ^*e*^Mann-Whitney U-test

When the polyunsaturated fatty acids were examined, there was no difference between the groups in the proportions of arachidonic acid, eicosapentaenoic acid, and docosahexaenoic acid in the lipoprotein fractions. However, the mean percentage of docosapentaenoic acid in LDL, HDL_2 _and HDL_3 _was significantly higher in the group of subjects with the higher MMP-9 level in comparison with those with the lower serum MMP-9 concentration (Table 2). Furthermore, the ratio of docosapentaenoic acid to arachidonic acid in LDL, HDL_2 _and HDL_3 _was higher in subjects with higher MMP-9 than in those with lower MMP-9 (p < 0.01 for all, data not shown).

## Discussion

Our result of a positive association between MMP-9 and the percentage of docosapentaenoic acid in isolated lipids of HDL and LDL may reflect increased metabolism of long chain fatty acids in subclinical inflammation, analogous to the situation seen in chronic non-active inflammatory bowel disease [12-15]. Our subjects were subjectively healthy, and the cause of variation of serum MMP-9 concentration remains unknown.

Docosapentenoic acid is one of the three major n-3 long chain polyunsaturated fatty acids in fish and marine oils. In addition to diet, eicosapentaenoic acid, docosapentaenoic acid, and docosahexaenoic acid are obtained by synthesis in the body from α-linolenic acid (18:3n-3) through a series of desaturation and elongation reactions. There is a possible explanation why increased synthesis of n-3 fatty acids could lead to increase in docosapentaenoic acid and not docosahexaenoic acid. It seems that docosapentaenoic acid is not easily further metabolized to docosahexaenoic acid in the human body because the pathway requires a rate-limiting Δ-6 desaturase reaction, chain elongation and translocation of the resulting 24:6n-3 to peroxisomes for oxidation [19]. N-3 fatty acids are often treated as a unity, and the effects of docosapentaenoic acid are rarely compared with those of eicosapentaenoic acid, and docosahexaenoic acid. Therefore the functions of docosapentaenoic acid are more poorly known.

Dietary supplementation with n-3 fatty acids has thought to be of benefit in the management of inflammatory diseases, because several studies have shown that n-3 fatty acids can inhibit the synthesis and release of proinflammatory cytokines such as tumor necrosis factor alpha and interleukin-1-β that are produced during inflammation [20]. These cytokines increase MMP-9 synthesis and activation by inflammatory cells [21]. Therefore, it is possible that a chronic inflammatory condition through increased utilization of anti-inflammatory n-3 fatty acids leads to increased activity of the biosynthesis of n-3 fatty acids. These changes would be seen in all lipoprotein classes through exchange of lipids.

## Conclusion

In conclusion, our results indicate that there is a positive correlation between n-3 docosapentaenoic acid and MMP-9, a potential marker of inflammation. What had triggered the rise in MMP-9 is not known. Together with previous studies, our findings suggest an increased biosynthesis of n-3 polyunsaturated fatty acids in subclinical inflammation.

## Methods

### Subjects

A total of 59 subjectively healthy 20 to 60 year-old women (n = 32) and men (n = 27) were recruited amongst the personnel and students of Medical School of the University of Tampere and Tampere University Hospital. All participants filled a questionnaire, where emphasis was given to their health status (diseases and use of medication) in addition to health related behavior (smoking, use of alcohol and vitamins). All participants gave their written consents to the study. The study protocol was approved by the ethics committee of the Tampere University Hospital.

### Laboratory methods

Blood samples were taken from the antecubital vein into suitable tubes (Vacuette, Greiner) using minimal stasis after a 12-hour fast while the subjects were seated (after a 15-min rest). Samples for the isolation of lipoproteins were taken into EDTA-containing tubes, immediately placed in ice, and plasma was separated after centrifugation (Heraeus, 2000 × g, + 4°C). EDTA-plasmas were supplemented with sucrose (0.6% w/v final concentration). All samples were kept frozen at -70°C until analyzed. Fasting blood glucose concentration was determined from capillary blood using Hemocue Glucose Analyzer (Hemocue, Ängelholm Sweden).

Plasma cholesterol, HDL cholesterol, triacylglycerol, apoA-I and apoB concentrations were measured with Cobas Integra 700 automatic analyzer using reagents and calibrators as recommended by the manufacturer (Roche Diagnostic, Basel, Schwitzerland). LDL cholesterol was calculated according to Friedewald.

For the accurate assessment of serum MMP-9, aliquots of sera were removed and stored at -70°C in a freezer that was not in daily use until analysis. Quantification of immunoreactive MMP-9 was carried out by enzyme-linked immunosorbent assay (ELISA) (Diabor Ltd, Oulu, Finland). ELISAs were performed on 96-well microtiter plates using standard protocols. Recombinant MMP-9 was used as standard. The microtiter plate was coated with the monoclonal antibody (code GE-213). The bound proteins from serum and standards were detected with a secondary polyclonal antibody produced in chicken against MMP-9. A peroxidase-labeled anti-chicken-IgG (Chemicon, USA) was used for detection of the bound secondary antibody. O-phenylenediamine (OPD) was used to visualize the peroxidase label. The color formation was measured at 450 nm (Anhos 2000 microplate reader) and calculations were done using a Multicalc program (Wallac, Turku, Finland). The monoclonal antibody recognized both the free MMP-9 and that bound to its inhibitor, tissue inhibitor of metalloproteinases-1 (TIMP-1) [2,18].

Lipoproteins were fractionated by isopycnic density gradient ultracentifugation. Two ml of plasma was mixed with 4.0 ml of d 1.35 g/l NaCl/KBr solution in a 14 × 95 mm tube (Beckman, Palo Alto, USA) and then successively overlayered with 4.5 ml of a d 1.006 salt solution and 1.0 ml of distilled water. For centrifugation a Beckman SW40 Ti rotor, at 36000 rpm for 40 hours in a Beckman L60 centrifuge at 10°C was used. After ultracentrifugation the contents of the tubes were fractionated with an Isco gradient fractionator (Model 640, Lincoln, USA). The 280 nm absorbance of the effluent was continuously monitored with an Isco UA-5 absorbance detector. The different lipoproteins were well separated by the resulting slightly curving salt gradient. The fractions belonging to LDL, HDL_2 _and HDL_3 _were pooled on the basis of the absorbance curve.

The total fatty acid compositions of the ultracentrifugally isolated LDL, HDL_2 _and HDL_3 _particles were analyzed by gas-liquid chromatography. Lipids were extracted with chloroform/methanol, partitioned and the chloroform phase was dried under N_2_. The lipids were then hydrolyzed and transesterified with H_2_SO_4 _in dry methanol at 85°C for 2 h under N_2_. Following the addition of water, methyl esters of the fatty acids were extracted with petroleum ether and analyzed in a Shimadzu GC-14A gas chromatograph (Shimadzu Corporation, Kyoto, Japan) with a flame ionization detector using a Supelco SP 2560 capillary column (100 m, 0.25 mm I.D., 0.20 μm film thickness). The carrier gas was helium. The column temperature was 180°C for 15 min, then programmed to increase at 3°C/min to 230°C and held for 40 min. The individual fatty acids were identified with the aid of a standard mixtures of methyl esters (Lipid standards 189-15 and 189-17, Sigma). The areas were measured with a Shimadzu C-R4A Chromatopac Integrator and the results expressed as percentages of the sum of all fatty acids from 14:0 to 22:6n-3. As a control sample we used a pool of isolated HDL that was suitably diluted and kept frozen at -70°C. The inter assay coefficient of variation for the percentage of different fatty acids ranged from 0.3 to 4.4 %.

### Statistical analysis

Results are expressed as means ± standard deviation. Plasma triacylglycerol concentrations were used as their logarithms but reported as original results. Comparisons were conducted by Mann-Whitney U-test. Univariate associations between variables were analyzed using Spearman's correlation coefficients. The Statistica for Windows (version 5.1) software package (Statsoft Inc., Oklahoma, USA) was used for statistical analysis.

## Authors' contributions

TS and OJ conceived of the study, performed the statistical analyses and wrote the initial manuscript. MP, AS and AK carried out the laboratory analyses. MH designed the ELISA. TL, HJ and STN participated in the study design and coordination and helped to draft the manuscript. All authors read and approved the final manuscript.
